# Uncontrolled Eating during Pregnancy Predicts Fetal Growth: The Healthy Mom Zone Trial

**DOI:** 10.3390/nu11040899

**Published:** 2019-04-21

**Authors:** Jennifer S. Savage, Emily E. Hohman, Katherine M. McNitt, Abigail M. Pauley, Krista S. Leonard, Tricia Turner, Jaimey M. Pauli, Alison D. Gernand, Daniel E. Rivera, Danielle Symons Downs

**Affiliations:** 1 Center for Childhood Obesity Research, The Pennsylvania State University, University Park, State College, PA 16802, USA; eeh12@psu.edu (E.E.H.); kmm6054@psu.edu (K.M.M.); 2 Department of Nutritional Sciences, The Pennsylvania State University, University Park, State College, PA 16802, USA; adg14@psu.edu; 3 Exercise Psychology Laboratory, Department of Kinesiology, The Pennsylvania State University, University Park, State College, PA 16802, USA; amp34@psu.edu (A.M.P.); kbl5167@psu.edu (K.S.L.); dsd11@psu.edu (D.S.D.); 4 Diagnostic Medical Sonography, South Hills School of Business and Technology, State College, PA 16801, USA; tslturner3@gmail.com; 5 Department of Obstetrics and Gynecology, Penn State College of Medicine, Hershey, PA 17033, USA; jpauli@pennstatehealth.psu.edu; 6 Department of Maternal & Fetal Medicine, Penn State College of Medicine, Hershey, PA 17033, USA; 7 Control Systems Engineering Laboratory, School for Engineering of Matter, Transport, and Energy, Arizona State University, Tempe, AZ 85287, USA; daniel.rivera@asu.edu

**Keywords:** pregnancy, gestational weight gain intervention, eating behavior, restraint, disinhibition, uncontrolled and emotional eating, fetal growth, overweight and obesity, generalized linear models

## Abstract

Excess maternal weight gain during pregnancy elevates infants’ risk for macrosomia and early-onset obesity. Eating behavior is also related to weight gain, but the relationship to fetal growth is unclear. We examined whether Healthy Mom Zone, an individually tailored, adaptive gestational weight gain intervention, and maternal eating behaviors affected fetal growth in pregnant women (*n* = 27) with a BMI > 24. At study enrollment (6–13 weeks gestation) and monthly thereafter, the Three-Factor Eating Questionnaire was completed. Ultrasounds were obtained monthly from 14–34 weeks gestation. Data were analyzed using multilevel modeling. Higher baseline levels of uncontrolled eating predicted faster rates of fetal growth in late gestation. Cognitive restraint was not associated with fetal growth, but moderated the effect of uncontrolled eating on fetal growth. Emotional eating was not associated with fetal growth. Among women with higher baseline levels of uncontrolled eating, fetuses of women in the control group grew faster and were larger in later gestation than those in the intervention group (study group × baseline uncontrolled eating × gestational week interaction, *p* = 0.03). This is one of the first intervention studies to use an individually tailored, adaptive design to manage weight gain in pregnancy to demonstrate potential effects on fetal growth. Results also suggest that it may be important to develop intervention content and strategies specific to pregnant women with high vs. low levels of disinhibited eating.

## 1. Introduction

Over 50% of women in the United States enter pregnancy already having overweight or obesity; the majority of these women (60%) gain more weight in pregnancy than is recommended by the Institute of Medicine (IOM) [1]. Data also indicate that 23% of US 2–5-year-olds have overweight [2] and 9%–14% have obesity [3,4], underscoring the need for research on early obesity prevention. The prenatal period may be an opportune time to intervene and break the intergenerational cycle of obesity by reducing fetal exposure to an “obesogenic” intrauterine environment [5,6] through promoting maternal energy balance (EB) (i.e., a nutrient-rich, low-energy dense diet and physical activity) [7]. Infant birth weight is commonly used as a surrogate marker for intrauterine growth and as an indicator of the conditions experienced in utero [8]. Maternal weight gain during pregnancy is necessary for healthy fetal development, but high gestational weight gain elevates infant risk for macrosomia and early onset obesity [1]. Further, infant birth weight is a positive, independent predictor of obesity during later childhood [9,10,11], and high birth weight is associated with increased risk for adult-onset obesity and diabetes [12].

Behavioral interventions have effectively managed weight among normal weight women, but similar interventions have had mixed effects among women with overweight or obesity [13]. Together, these data indicate the need for effective interventions that promote healthy gestational weight gain and are able to influence the etiology of obesity for offspring at a critical time in development. Based on past research and our own pilot data [7,14,15,16,17], we developed Healthy Mom Zone [18], an individually tailored and “intensively adaptive” intervention to effectively manage gestational weight gain and promote optimal maternal and child health in women with overweight or obesity. This novel intervention provides each woman with the support needed to manage her gestational weight gain, while adapting the dose and intensity of the intervention to her unique needs across pregnancy. Healthy Mom Zone is also innovative, applying principles/methods from systems science and engineering [19] including dynamical modeling, simulation, and controller design to optimize the intervention. Intensive longitudinal data (e.g., weight, energy intake, eating behavior, physical activity, and psychosocial factors) were collected and used in making decisions about adapting the intervention.

Throughout pregnancy, maternal nutritional status and dietary intake are key factors influencing the health of both the child and mother [20]. However, misreporting of dietary intake is common, making it difficult to identify how dietary intake patterns, macronutrient intakes, or even energy density are associated with gestational weight gain and fetal programming [21]. Among pregnant women with overweight or obesity, as many as one-third to one-half under-report dietary intake, with rates higher in late compared to early pregnancy [22], a finding supported by our own Healthy Mom Zone data [23].

Moreover, an individual’s eating behavior and attitudes influence food choices, impact the regulation of food intake, and ultimately affect weight. Given the recall bias of self-reported dietary intake, alternative modifiable factors such as eating behavior (e.g., restrained eating, uncontrolled eating, emotional eating) that are relatively easy to measure may be amenable to intervention during pregnancy. Dietary restraint and disinhibited eating (emotional and uncontrolled), as measured by the Eating Inventory (EI) (also referred to as the Three Factor Eating Questionnaire [24,25]), are psychological constructs that assess behavioral control and attitudes toward food and eating. Cognitive dietary restraint is defined as a tendency to consciously restrict or control food intake, regardless of physiological signs of hunger and satiety. Uncontrolled eating is defined as a tendency to overeat relative to physiologic need and feeling of lack of control, whereas emotional eating is the tendency to overeat during depressed and melancholic states [26].

Eating behaviors may be a “proxy” for dietary intake in lieu of accurate, self-reported dietary intake [27]. In the general population, disinhibited eating is positively associated with food intake and weight [28,29,30,31,32], but the association between dietary restraint and emotional eating with these outcomes remains unclear [29,30,31,33,34,35,36,37]. In our current obesogenic environment, cognitive controls of eating may be necessary to prevent overeating and moderate weight gain [38]. For example, a randomized weight loss study reported that cognitive restrained eating was associated with lower energy intakes [39]. In pregnant women, restrained eating and emotional eating have been positively associated with poorer diet quality and greater overall weight gain [40,41,42,43]. However, cross-sectional studies [30,31,44] and longitudinal data [45] in nonpregnant individuals have revealed that it is not the independent effects of restraint and disinhibition but their interaction that predicts food intake and ultimately body weight, with restraint moderating the impact of uncontrolled eating. No study, to our knowledge, has examined the long-term effects of the interaction between restrained and disinhibited eating (uncontrolled or emotional) in pregnant women with overweight/obesity on fetal growth.

The primary aim of this study was to determine the effect of an individually tailored, adaptive intervention designed to help pregnant women with overweight and obesity to manage their gestational weight gain within the IOM guidelines on fetal growth, compared to a standard of care control. A secondary aim was to explore the interactive effects of maternal cognitive restraint, with uncontrolled and emotional eating during pregnancy, on fetal growth. Based on previous data [27,32,36,40,41,45], we expect that: (1) fetuses of women receiving the treatment would gain weight slower on average compared to control fetuses; (2) higher levels of emotional and uncontrolled eating will predict faster fetal weight gain; and (3) cognitive restraint will attenuate the positive relationship between emotional and uncontrolled eating and fetal weight gain. Findings from this study may inform counseling during pregnancy.

## 2. Methods

### 2.1. Study Design and Participants

Data for this analysis were collected as part of an ancillary study to measure fetal growth in women participating in Healthy Mom Zone, an optimization trial within the multiphase optimization strategy (MOST) framework [46], of an adaptive intervention to manage gestational weight gain among pregnant women with overweight or obesity [18]. This study was approved by the Pennsylvania State University Institutional Review Board (study ID #00003752, initial approval date 12/1/2015), and participants provided written consent for their participation. Women were eligible if they were 6–13 weeks pregnant with a single fetus, English-speaking, non-smoking, had a prepregnancy BMI > 24, and were free of significant pregnancy complications or medical conditions. The study was powered on the primary outcome of gestational weight gain; a sample size of 24 participants (12 per group) was determined to yield 80% power to detect a standardized effect size for gestational weight gain of 1.2 using a two-sided test with a significance level of *p* = 0.05. We aimed to recruit 30 participants, accounting for up to 25% drop out [18]. Participants (*n* = 31) were randomized to the intervention or a standard care control group. From this initial group, three women had a miscarriage at <12 weeks gestation, and one woman dropped out of the study at 22 weeks gestation, representing 87.1% sample retention (9.7% miscarriage rate and 3.2% drop rate). For the purpose of analysis, women with a prepregnancy BMI 24.1–29.9 were classified as having overweight (OW) and those with BMI ≥ 30 were classified as having obesity (OB). Fetal growth data were collected on a total of 27 women (intervention: 9 OW/4 OB; control: 7 OW/7 OB). One woman (control group, OW) did not complete the baseline survey measure of eating behavior, resulting in a sample of *n* = 26 women for those analyses.

### 2.2. Intervention

Healthy Mom Zone is an individually tailored, adaptive intervention to optimize gestational weight gain among pregnant women with overweight and obesity. This optimization trial was built within the multiphase optimization strategy (MOST) framework [46] with the aim of developing an intervention to efficiently and effectively manage weight gain over pregnancy. Details of the Healthy Mom Zone intervention are published elsewhere [18], but in short, principles from the theory of planned behavior (TPB) [47] and self-regulation [48] provide the conceptual framework for the intervention. The key intervention components include education, goal-setting/action plans, self-monitoring, and active learning. Participants in the intervention group met weekly with a study dietitian and were given customized calorie and physical activity goals. Plots of each woman’s weight, physical activity, and dietary intake via real-time data collection procedures were plotted to generate a report illustrating gestational weight gain in the context of the IOM guidelines. This report was discussed by the dietitian. In addition, emails were sent discussing maternal behaviors that impact baby health and growth. Lastly, gestational weight gain was evaluated weekly and decision rules were used in 3–4 week cycles to decide when and how the intervention may be adapted for an individual. When a participant’s weight gain trajectory exceeded the recommended rate, they received an intervention step-up consisting of additional healthy eating active learning sessions (e.g., cooking demonstrations, grocery store tours) and onsite physical activity sessions with a fitness instructor. All women in the study received standard prenatal care (e.g., regular visits, prenatal counseling) with their personal healthcare provider.

### 2.3. Measures

At study enrollment, height and weight were measured by clinical research center staff. Participants completed a baseline background questionnaire that assessed prepregnancy weight, height, age, years of education, combined family income, and general health. At study enrollment and every four weeks thereafter, women completed the three-factor eating questionnaire (TFEQ-21) [24] via online survey through REDCap [49]. This 21-item scale, with a four point response scale ranging from (1) definitely true to (4) definitely false, measures three subscales: cognitive restraint (e.g., “I consciously hold back on how much I eat at meals to keep from gaining weight.”), uncontrolled eating (e.g., “Sometimes when I start eating, I just can’t seem to stop.”), and emotional eating (e.g., “I start to eat when I feel anxious.”). Scores for each subscale were calculated by averaging respective items.

Standard ultrasounds were obtained at approximately 14, 18, 22, 26, 30, and 34 weeks gestation by a sonographer using the same ultrasound machine (Philips iU22 MATRIX, Philips Healthcare, Andover, MA, USA). Standard fetal biometry measures including biparietal diameter (BPD), head circumference (HC), abdominal (AC), and femur length (FL), were assessed. Estimated fetal weight was calculated using a validated equation [50]. Gestational age was calculated using the date of last menstrual period. All ultrasounds were reviewed by a study physician, and any concerns identified were communicated to the participant’s personal physician. Birth weight and gestational age at birth were abstracted from medical records or reported by participants if medical records were not available. Sex-specific birth weight-for-gestational age percentiles were determined using the INTERGROWTH-21st standards [51].

### 2.4. Analysis

Eating behavior variables were centered to disaggregate within- and between-person effects and to aid interpretation. Between-person effects were assessed using baseline levels of each eating behavior centered on the population mean, so that the centered baseline values represented each individual’s deviation from the average baseline level across all women in the study. Within-person effects were assessed using change scores, such that each monthly eating behavior score represented a woman’s change from her own baseline. Though survey data (i.e., eating behavior) and fetal ultrasounds were collected monthly, the timing of these measurements relative to one another was variable due to variation in gestational age at enrollment. For the purpose of this analysis, ultrasound data was paired with the most recent eating behavior survey completed prior to the scan. In seven instances across four participants, no eating behavior data preceded the scan, thus, survey data completed within seven days after the scan were used. The mean (SD) time between survey completion and ultrasound scan was 12.2 (8.9) days.

Statistical analysis was performed in SAS 9.4 (SAS Institute, Cary, NC, USA). Data were analyzed using multilevel modeling (PROC MIXED). All models utilized the restricted maximum likelihood (REML) estimation method, Satterthwaite method for computing df, and an unstructured covariance structure. Determination of model fit was based on several criteria including model convergence, a positive definite G matrix, and Akaike information criteria. Intraclass correlation coefficients (ICCs) were calculated as the ratio of between-subjects variance to total variance. Change over time in each of the three eating behavior subscales was modeled as a linear function of gestational week with a random intercept and slope. For cognitive restraint, the random gestational week slope term resulted in a nonpositive definite G matrix, so it was removed from the model. Interactions of gestational week with study group and prepregnancy BMI were tested to determine if the rate of change over time differed by these variables.

Estimated fetal weight was also modeled using multilevel modeling. Linear, quadratic, and cubic effects of gestational week were considered. Once the model describing fetal weight over time was finalized, study group and maternal prepregnancy BMI were added, and interactions with gestational week were tested. Next, a set of models were run for each of the three eating behavior constructs. These models tested (a) main effect of the baseline eating behavior, (b) interaction between baseline eating behavior and gestational week, (c) main effect of change in eating behavior, (d) interaction between change in eating behavior and gestational week, (e) interaction between baseline and change in eating behavior, and (f) three-way interaction between baseline and change in eating behavior and gestational week. For all interactions with gestational week, interactions with both the linear and quadratic gestational week terms were tested; the interaction with the quadratic term was dropped when not significant and was not reported here. Finally, two sets of models were tested to evaluate interactions between cognitive restraint and each of the two disinhibited eating behavior constructs (uncontrolled eating and emotional eating). These models tested (a) interaction between baseline cognitive restraint and baseline disinhibited eating; (b) three-way interactions between baseline cognitive restraint, baseline disinhibited eating, and gestational week; (c) interactions of baseline cognitive restraint and disinhibited eating with change in cognitive restraint and disinhibited eating; (d) three-way interactions between change in cognitive restraint, change in disinhibited eating, and gestational week; and (e) interactions described in (a)–(d) but with the quadratic gestational age term in place of the linear term. Two final models, one including cognitive restraint × uncontrolled eating and one including cognitive restraint × emotional eating, were evaluated using backward and forward selections.

## 3. Results

### 3.1. Descriptive Characteristics

Descriptive statistics are located in Table 1. Mean age of women at study entry was 30.6 (SD = 2.9) ranging from 24 to 37 years. Mean gestational age at the start of the study was 10.6 weeks (median 10.7 weeks). The majority of women were married and pregnant with their first baby (66.7% no prior live births; 33.3% had 1 previous live birth). Women were, on average, well-educated and fairly affluent. Moreover, the majority (81.5%) of women were employed full-time. Forty-one percent of women had obesity prior to pregnancy. Infants were born at a mean of 39.5 (1.4) weeks gestation. Mean birth weight was 3381 g (SD 617, range 2050–4570), reflecting an average birth weight for gestational age percentile of 56.6 (SD 31.9, range 2.6–99.4). Three infants (11.1%, 2 intervention, 1 control) were macrosomic (birth weight > 4000 g), and four infants (14.8%, 2 intervention, 2 control) were small for gestational age (SGA, <10th percentile) at birth. Out of 162 ultrasound scans, eight (4.9%) were flagged by the study physician, including five for growth restriction concerns. For two of these participants (both intervention), the concern resolved on its own at subsequent scans.

Mean (SD) baseline values were 2.79 (0.47) for cognitive restraint (range = 2.00–3.67), 2.15 (0.39) for uncontrolled eating (range = 1.33–3.00), and 2.14 (0.59) for emotional eating (range = 1.00–3.33). Baseline values did not differ by study group. Baseline cognitive restraint and uncontrolled eating did not differ by prepregnancy BMI status, but women with obesity had significantly higher baseline emotional eating than those with overweight (2.41 (0.52) vs. 1.95 (0.57), *p* = 0.047). Cognitive restraint significantly decreased across gestation (restraint = 2.90–0.014 × gestational age in weeks, *p* = 0.0008), as did uncontrolled eating (uncontrolled eating = 2.25–0.007 × gestational age in weeks, *p* = 0.006). Emotional eating did not significantly change across gestation (emotional eating = 2.10–0.006 × gestational age in weeks, *p* = 0.14), and there were no differences in rate of change by study group or prepregnancy BMI status for any of the eating behavior subscales. Baseline cognitive restraint was negatively associated with change in cognitive restraint (*p* = 0.03), such that women with higher baseline restraint experienced greater decreases in restraint across pregnancy. However, there was no association between baseline scores and change scores for uncontrolled eating and emotional eating. Intraclass correlations were 0.59 for cognitive restraint, 0.85 for uncontrolled eating, and 0.79 for emotional eating, suggesting that the majority of variance in all three constructs was between individuals rather than within individuals.

### 3.2. Unconditional Polynomial Models for Fetal Weight Change

Fetal growth from 14–34 weeks was modeled using a multilevel model. The first model included a random intercept and random and fixed linear effects of gestational week. The random intercept resulted in a nonpositive definite G matrix and was removed from the model. Next, a quadratic fixed effect of gestational week was added, which improved the fit of the model (likelihood ratio test *p* < 0.0001). Quadratic random effects and cubic fixed and random effects were tested but did not improve model fit.

### 3.3. Conditional Polynomial Models for Fetal Weight Change

#### 3.3.1. Treatment Effect of Intervention and Fetal Growth

**Figure 1:**
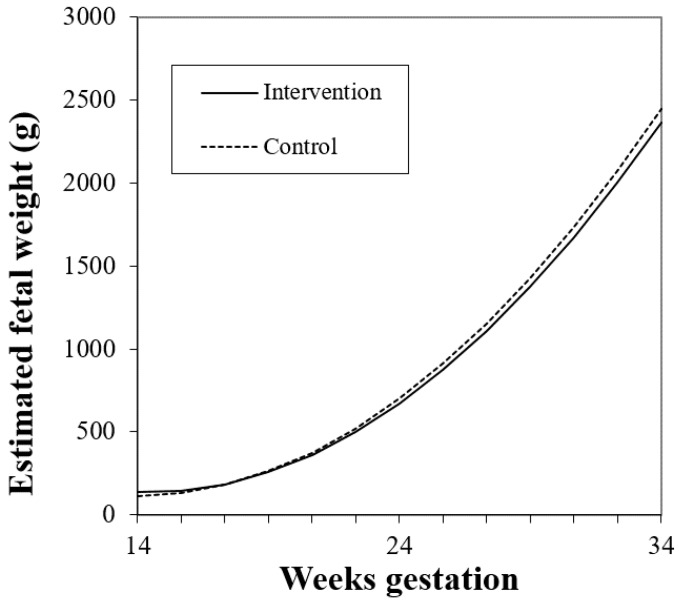
Estimated fetal weight trajectory in intervention and control group (study group × gestational week, *p* = 0.095). Estimates were generated using multilevel modeling (SAS PROC MIXED).

For Model 1, there was no significant main effect of study group on fetal weight. There was a trend for an interaction between study group and gestational week (*p* = 0.095, Model 1, Table 2), such that fetuses of women in the control group tended to grow faster and be larger in later gestation than those in the intervention group (Figure 1), before and after adjusting for prepregnancy BMI.

#### 3.3.2. Individual Eating Behaviors and Fetal Growth

**Figure 2:**
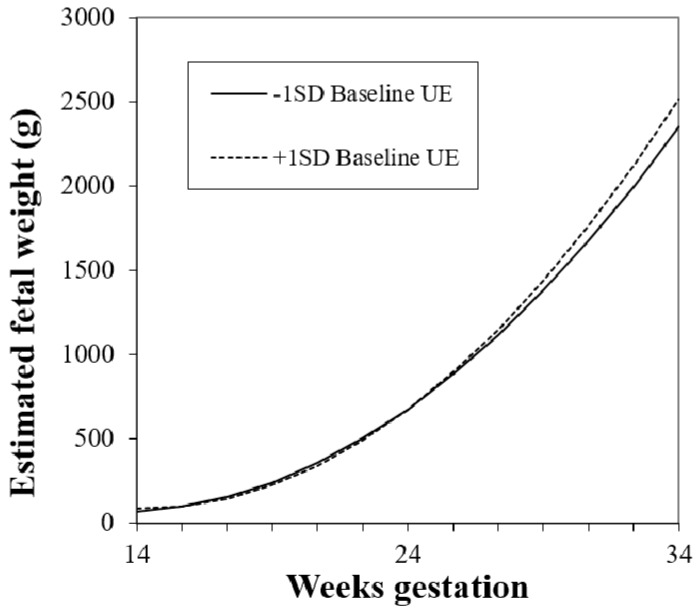
Estimated fetal weight trajectory at higher (mean + 1 SD) and lower (mean – 1 SD) levels of baseline uncontrolled eating (UE), (baseline uncontrolled eating × gestational age^2^, *p* = 0.03). Estimates were generated using multilevel modeling (SAS PROC MIXED).

Eating characteristics (uncontrolled eating, emotional eating, and cognitive restraint) were each added, individually, to Model 1 to test higher order interactions and main effects. When higher-order interactions were not significant, models were simplified. For uncontrolled eating, there was a significant two-way interaction between baseline uncontrolled eating and gestational age^2^ (Table 3, Model 2, *p* = 0.03). As shown in Figure 2, women with higher baseline levels of uncontrolled eating had faster rates of fetal growth in late gestation. Next, change in uncontrolled eating from baseline was added to the model. There was a significant interaction between change in uncontrolled eating and gestational age (Table 4, Model 3, *p* = 0.04). In contrast to the effect of baseline uncontrolled eating, women who experienced greater decreases in uncontrolled eating had faster rates of fetal growth. In both Models 2 and 3, the gestational week × study group effect was statistically significant. In addition, the interaction between baseline uncontrolled eating and gestational week^2^ from Model 2 remained statistically significant with the addition of change in uncontrolled eating, suggesting independent effects of baseline and change in this eating behavior. Neither baseline levels nor change across gestation in cognitive restraint or emotional eating were associated with fetal growth.

#### 3.3.3. Cognitive Restraint × Disinhibited Eating and Fetal Growth

Lastly, backward and forward selection were used to estimate a final model that included interactions between cognitive restraint and disinhibited eating (i.e., emotional eating and uncontrolled eating). All potential three-way interactions were considered. For cognitive restraint × uncontrolled eating (Table 5, Model 4), there were no significant three-way interactions. Baseline cognitive restraint × change in uncontrolled eating emerged as significant (*p* = 0.02). In women who experienced increases in uncontrolled eating across pregnancy, baseline cognitive restraint was negatively associated with fetal weight. This interaction was not specific to a particular time in gestation (i.e., there was no significant three-way interaction between baseline cognitive restraint, change in uncontrolled eating, and gestational week). The interaction between baseline uncontrolled eating and gestational age^2^ remained significant, such that women with higher baseline levels of uncontrolled eating had faster rates of fetal growth in late gestation. However, the interaction between change in uncontrolled eating and gestational age observed in Model 3 did not emerge as significant in this final model. Lastly, the effect of study group on change in fetal growth remained significant (study group × gestational week *p* = 0.02).

For cognitive restraint × emotional eating, there were no statistically significant main effects or interactions between restraint and emotional eating.

#### 3.3.4. Post-hoc Analysis: Study Group × Uncontrolled Eating Interaction

**Figure 3:**
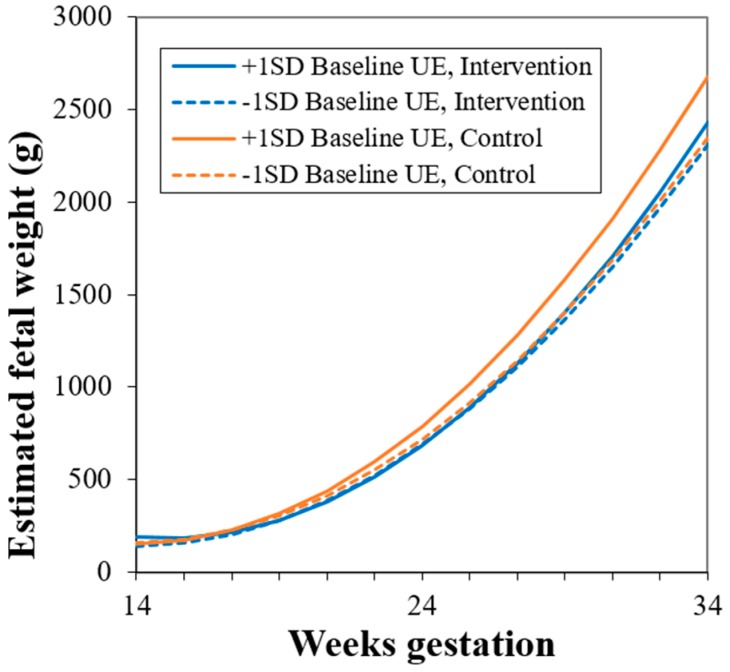
Estimated fetal weight trajectory at higher (mean + 1 SD) and lower (mean – 1 SD) levels of baseline uncontrolled eating (UE) in intervention and control group participants, (baseline uncontrolled eating × study group × gestational age, *p* = 0.04). Estimates were generated using multilevel modeling (SAS PROC MIXED).

Although neither baseline nor change in uncontrolled eating differed by study group, inclusion of uncontrolled eating in Models 2–4 resulted in a statistically significant interaction between study group and gestational age. To further investigate why this relationship might emerge after accounting for baseline uncontrolled eating, we explored adding interactions between uncontrolled eating and study group to Model 4. A two-way interaction between uncontrolled eating and study group was not statistically significant, but there was evidence for a three-way interaction between baseline uncontrolled eating, study group, and gestational age (*p* = 0.02, Model 5, Table 6). Higher baseline level of uncontrolled eating was associated with faster fetal growth among control group participants but not among those receiving the intervention (Figure 3). Similar results were observed when these interactions were added to Models 2 and 3.

## 4. Discussion

Healthy Mom Zone, an individually tailored, adaptive intervention designed to effectively manage gestational weight gain among women with overweight or obesity tended to result in a slower rate of fetal growth compared to fetuses of control women, with significant effects emerging in models that accounted for uncontrolled eating. Higher uncontrolled eating at study entry was associated with faster fetal growth. Findings also suggest that cognitive restraint on its own appears to have little effect on fetal growth, but may help moderate fetal growth among women who experience increases in uncontrolled eating during pregnancy. Several of these findings warrant discussion.

Though not statistically significant in Model 1, the Healthy Mom Zone intervention had a protective effect on fetal growth once uncontrolled eating was added (Models 2–4); intervention fetuses gained weight at a slower rate on average compared to control fetuses. It is important to note that the intervention did not increase risk for an infant being born small for gestational age. This finding has clinical relevance for several reasons. Women who are overweight or obese are 65% and 163% more likely, respectively, to deliver babies who are large for their gestational age, regardless of whether the mother develops gestational diabetes during pregnancy [52]. In addition, risk of fetal macrosomia, having a birth weight of more than 8 pounds or 4000 grams regardless of gestational age, is more likely among women with obesity. This is important because women with overweight or obesity are also more likely to exceed gestational weight gain guidelines, further elevating infant risk for macrosomia. The clinical relevance is that decreasing risk for faster weight gain may decrease health risks for the mother (e.g., labor and delivery complications) and the infant (e.g., childhood obesity, metabolic syndrome) both during pregnancy and after childbirth. Thus, our behavioral intervention has the potential to mediate this risk, in particular among pregnant women with overweight or obesity. 

In the current study, both cognitive restraint and uncontrolled eating decreased on average across gestation, and there was no change in emotional eating. Similarly, previous studies have found that cognitive restraint is lower during pregnancy compared to prepregnancy levels or non-pregnant women [53,54]. Research on changes in disinhibition across pregnancy is scant. We did not observe any association between prepregnancy BMI and cognitive restraint or uncontrolled eating; however, this may be because our sample was limited to women with BMI > 24. Two previous studies have found that pregnant women with obesity tend to report higher levels of disinhibited eating [55,56] than their normal weight counterparts. Further research is needed to evaluate how these eating behaviors change throughout pregnancy.

As hypothesized, greater uncontrolled eating in late first trimester was associated with faster fetal growth in late pregnancy. However, there was no association between emotional eating and fetal growth. To our knowledge, this study is the first to describe relationships between maternal disinhibited eating during pregnancy and fetal growth in utero in a non-eating disorder population. One previous study has examined the relationship between maternal eating behavior and postnatal growth. Mothers’ emotional eating, assessed at infant age 12 months, was positively associated with infant weight gain through 12 months, while external eating was negatively associated with infant weight gain. Maternal restraint was not associated with infant weight gain [57]. Further research is needed to clarify the relationship between maternal disinhibited eating and fetal/infant weight gain, and to determine the extent to which this relationship is reflective of modifiable factors, such as maternal diet, weight gain, and feeding practices, as opposed to nonmodifiable factors such as genetics.

Emotional eating and external eating have been associated with excess gestational weight gain [42]. Qualitative studies of pregnant women have found that emotional and external eating are often described as a barrier to healthy eating and management of gestational weight gain [58,59,60]. Our finding that uncontrolled eating is associated with fetal weight gain adds to this literature, suggesting that disinhibited eating behaviors may be an ideal target for gestational weight gain management interventions. Our Healthy Mom Zone intervention focused primarily on dietary intake, physical activity, and self-monitoring of weight. Expansion of intervention content focused on mindfulness, stress reduction, and emotion regulation, strategies that have been shown to reduce disinhibited eating in nonpregnant populations [61,62,63], may further benefit pregnant women with overweight or obesity.

Similar to the uncertain relationship between restraint and weight in nonpregnant adults, mixed findings have been reported for restraint and gestational weight gain [41,42,64,65]. Previous work in nonpregnant populations suggests that restraint may not be directly associated with weight but rather as a moderator of the effect of disinhibition [30,31,44,45]. A similar interaction effect may be involved in fetal growth. We observed a significant interaction between baseline cognitive restraint and change in uncontrolled eating, where among women who experienced increases in uncontrolled eating during pregnancy, those with higher baseline cognitive restraint had smaller fetuses than those with lower baseline cognitive restraint [31,44,45]. This suggests that a greater ability to consciously control food intake may help protect against adverse effects of increases in uncontrolled eating on fetal weight gain.

We did not have an a priori hypothesis about differential effects of the intervention on fetal growth by baseline eating behavior, but our post-hoc analysis of the interaction between study group and baseline uncontrolled eating suggests that the intervention may have been beneficial, particularly for women with higher baseline uncontrolled eating. It is possible that the intervention provided women who were already prone to uncontrolled eating with strategies to cope with inclinations to overeat, providing a buffering effect against excess fetal growth. Future analyses will examine whether baseline levels of eating behavior predict differential response to the Healthy Mom Zone intervention for other outcomes including maternal weight gain. Factors such as eating behavior that predict intervention response may be used to identify patients that are most likely to benefit from an intensive intervention.

There are also some limitations of this research. The small sample size, although adequate for an optimization trial within the MOST framework [46], precludes the ability to make assumptions at a population level. The intervention and control groups were unbalanced in terms of the proportion of women with obesity; however, all analyses controlled for prepregnancy BMI. In addition, the target population was a homogenous sample of women (e.g., married, middle to upper class, Caucasian) from largely rural and suburban areas in central Pennsylvania, thus limiting the extension of the study findings to more culturally diverse and urban populations of pregnant women with overweight or obesity. Future research will aim to broaden generalizability of our study findings. In addition, we do plan to understand the application of Healthy Mom Zone to a more diverse sample of pregnant women in the future, including those who are normal weight, more culturally diverse, and reside in varied communities across the US. Lastly, intensive longitudinal data collection may have affected behavior, and responses may have become biased due to repeated assessments throughout gestation. However, this longitudinal design is also a strength of this study, allowing us to better understand how individual differences in eating behavior are related to fetal growth.

## 5. Conclusions

Healthy Mom Zone, an individually tailored, intensive behavioral intervention designed to help women with overweight or obesity manage gestational weight gain shows promise for preventing rapid fetal growth, particularly for women prone to uncontrolled eating, though these findings must be replicated in a larger and more diverse sample. Our results also suggest that uncontrolled eating may increase risk for faster fetal growth, and that restraint on its own seems to have little influence. However, among women who experience increases in uncontrolled eating during pregnancy, higher restraint may help moderate fetal growth. Implications for these findings include that understanding participant eating behaviors at baseline may be important for future gestational weight gain interventions. Lastly, findings illustrate a need to develop intervention content and strategies specific to women with high vs. low levels of disinhibited eating.

## Figures and Tables

**Table 1:** Characteristics of women at baseline (*n* = 27).

	Overall (*n* = 27)	Intervention (*n* = 13)	Control (*n* = 14)
	M (SD) Range	M (SD) Range	M (SD) Range
Age (years)	30.6 (2.9) 24–37	30.3 (2.8) 24–35	30.9 (3.2) 27–37
Gestational Age at Study Start (weeks)	10.6 (1.6) 7.3–13.0	10.5 (1.7) 7.3–13.0	10.6 (1.6) 7.9–12.9
Weight at Study Start (lbs.)	190.4 (46.8) 134–294	182.2 (40.5) 145–294	197.9 (52.3) 134–292
Body Mass Index	31.6 (7.0) 24.1–48.9	30.7 (6.7) 24.1–48.9	32.4 (7.5) 24.5–42.8
	%	%	%
% BMI = 24.1–29.9	59.3	69.2	50
% Obese (BMI ≥ 30)	40.7	30.8	50
Parity			
Primiparous (no prior live birth)	66.7	53.9	78.6
Multiparous: 1 previous live birth	33.3	46.2	21.4
Education			
Graduate/professional	48.2	38.5	57.1
College	48.2	61.5	35.7
High school	3.7	0	7.1
Marital Status			
Married	92.6	92.3	92.9
Single	3.7	0	7.1
Divorced	3.7	7.7	0
Race			
Non-Hispanic White	96.3	92.3	100
Asian	3.7	7.7	0
Family Income			
>$100,000	33.3	30.8	35.7
$40–$100,000	44.4	61.5	28.6
$20–$40,000	18.5	0	35.7
$10–$20,000	3.7	7.7	0
Employment			
Full-time	81.5	84.6	78.6
Part-time	11.1	15.4	7.1
Self-employed	3.7	0	7.1
Other	3.7	0	7.1

Abbreviations: M = Mean; SD = Standard deviation; BMI = body mass index.

**Table 2:** Multilevel model parameter estimates predicting change in fetal weight (g) based on randomized intervention vs. control and interactions with eating (*n* = 27).

	Model 1: Study Group		
Term	Est	SE	*p*
Intercept	1232.8	151.2	<0.0001
Gestational week	−161.9	10.34	<0.0001
Gestational week^2^	5.80	0.21	<0.0001
Prepregnancy BMI	0.30	2.74	0.91
Study group: Intervention *	89.46	61.56	0.14
Study group * × gestational week	−4.94	2.93	0.095

* Study group reference level is control. Abbreviations: Est = estimate; SE = standard error; BMI = body mass index.

**Table 3:** Multilevel model parameter estimates predicting change in fetal weight (g) based on randomized intervention vs. control and baseline uncontrolled eating (*n* = 26).

	Model 2: Study group + Baseline Uncontrolled Eating		
Term	Est	SE	*p*
Intercept	1195.7	154.5	<0.0001
Gestational week	−160.9	10.34	<0.0001
Gestational week^2^	5.80	0.21	<0.0001
Prepregnancy BMI	0.89	2.86	0.76
Study group: Intervention *	122.0	63.02	0.06
Study group * × gestational week	−6.59	3.03	0.03
Baseline uncontrolled eating	475.3	330.5	0.15
Baseline uncontrolled eating × gestational week	−49.04	28.21	0.08
Baseline uncontrolled eating × gestational week^2^	1.21	0.57	0.03

* Study group reference level is control. Abbreviations: Est = estimate; SE = standard error; BMI = body mass index.

**Table 4:** Multilevel model parameter estimates predicting change in fetal weight (g) based on randomized intervention vs. control, baseline uncontrolled eating, and change in uncontrolled eating (*n* = 26).

	Model 3: Study group + Baseline Uncontrolled Eating + Change in Uncontrolled Eating		
Term	Est	SE	*p*
Intercept	1270.8	154.3	<0.0001
Gestational week	−159.6	10.63	<0.0001
Gestational week^2^	5.74	0.21	<0.0001
Prepregnancy BMI	−1.45	2.71	0.60
Study group: Intervention *	110.3	64.65	0.09
Study group * × gestational week	−6.56	2.96	0.03
Baseline uncontrolled eating	532.6	330.8	0.11
Baseline uncontrolled eating × gestational week	−53.72	28.26	0.06
Baseline uncontrolled eating × gestational week^2^	1.32	0.57	0.02
Change in uncontrolled eating	252.1	181.0	0.17
Change in uncontrolled eating × gestational week	−13.94	6.74	0.04

* Study group reference level is control. Abbreviations: Est = estimate; SE = standard error; BMI = body mass index.

**Table 5:** Multilevel model parameter estimates predicting change in fetal weight (g) based on randomized intervention vs. control, uncontrolled eating, and cognitive restraint (*n* = 26).

	Model 4: Study Group + Uncontrolled Eating + Cognitive Restraint		
Term	Est	SE	*p*
Intercept	1283.4	156.4	<0.0001
Gestational week	−161.4	10.49	<0.0001
Gestational week^2^	5.80	0.21	<0.0001
Prepregnancy BMI	−1.28	2.92	0.66
Study group: Intervention *	113.2	64.17	0.08
Study group * × gestational week	−7.45	3.05	0.02
Baseline uncontrolled eating	501.5	328.3	0.13
Baseline uncontrolled eating × gestational week	−49.78	28.03	0.08
Baseline uncontrolled eating × gestational week^2^	1.25	0.56	0.03
Change in uncontrolled eating	−116.4	52.68	0.03
Baseline cognitive restraint	11.18	42.88	0.80
Baseline cognitive restraint × change in uncontrolled eating	−272.3	116.9	0.02

* Study group reference level is control. Abbreviations: Est = estimate; SE = standard error; BMI = body mass index.

**Table 6:** Multilevel model parameter estimates predicting change in fetal weight (g) including interactions between study group and uncontrolled eating (*n* = 26).

	Model 5: Study Group × Uncontrolled Eating + Cognitive Restraint		
Term	Est	SE	*p*
Intercept	1267.4	155.7	<0.0001
Gestational week	−160.6	10.40	<0.0001
Gestational week^2^	5.80	0.21	<0.0001
Prepregnancy BMI	−1.21	2.93	0.68
Study group: Intervention *	117.0	63.58	0.07
Study group * × gestational week	−7.67	3.03	0.01
Baseline uncontrolled eating	256.8	352.8	0.47
Baseline uncontrolled eating × gestational week	−35.22	28.64	0.22
Baseline uncontrolled eating × gestational week^2^	1.18	0.56	0.04
Change in uncontrolled eating	−111.3	52.60	0.04
Baseline cognitive restraint	10.36	42.70	0.81
Baseline cognitive restraint × change in uncontrolled eating	−259.0	116.1	0.03
Study group × baseline uncontrolled eating	310.6	175.3	0.08
Study group × baseline uncontrolled eating × gestational week	−17.00	8.25	0.04

* Study group reference level is control. Abbreviations: Est = estimate; SE = standard error; BMI = body mass index.

## References

[1] Institute of Medicine (US) and National Research Council (US) Committee to Reexamine IOM Pregnancy Weight Guidelines (2009). Weight Gain during Pregnancy: Reexamining the Guidelines.

[2] Ogden C.L., Carroll M.D., Kit B.K., Flegal K.M. (2014). Prevalence of childhood and adult obesity in the United States, 2011–2012. JAMA.

[3] Ogden C.L., Carroll M.D., Lawman H.G., Fryar C.D., Kruszon-Moran D., Kit B.K., Flegal K.M. (2016). Trends in Obesity Prevalence Among Children and Adolescents in the United States, 1988–1994 through 2013–2014. JAMA.

[4] Hales C.M., Fryar C.D., Carroll M.D., Freedman D.S., Ogden C.L. (2018). Trends in Obesity and Severe Obesity Prevalence in US Youth and Adults by Sex and Age, 2007–2008 to 2015–2016. JAMA.

[5] Patti M.E. (2013). Intergenerational programming of metabolic disease: Evidence from human populations and experimental animal models. Cell. Mol. Life Sci..

[6] Adamo K.B., Ferraro Z.M., Brett K.E. (2012). Can we modify the intrauterine environment to halt the intergenerational cycle of obesity?. Int. J. Environ. Res. Public Health.

[7] Dong Y., Rivera D.E., Thomas D.M., Navarro-Barrientos J.E., Downs D.S., Savage J.S., Collins L.M. A Dynamical Systems Model for Improving Gestational Weight Gain Behavioral Interventions. Proceedings of the American Control Conference.

[8] Fallucca S., Vasta M., Sciullo E., Balducci S., Fallucca F. (2009). Birth weight: Genetic and intrauterine environment in normal pregnancy. Diabetes Care.

[9] Reilly J.J., Armstrong J., Dorosty A.R., Emmett P.M., Ness A., Rogers I., Steer C., Sherriff A. (2005). Early life risk factors for obesity in childhood: Cohort study. BMJ.

[10] Martin J.A., Hamilton B.E., Ventura S.J., Osterman M.J., Kirmeyer S., Mathews T.J., Wilson E.C. (2011). Births: Final data for 2009. Natl. Vital Stat. Rep..

[11] Nohr E.A., Vaeth M., Baker J.L., Sorensen T.I., Olsen J., Rasmussen K.M. (2009). Pregnancy outcomes related to gestational weight gain in women defined by their body mass index, parity, height, and smoking status. Am. J. Clin. Nutr..

[12] Wei J.-N., Sung F.-C., Li C.-Y., Chang C.-H., Lin R.-S., Lin C.-C., Chiang C.-C., Chuang L.-M. (2003). Low birth weight and high birth weight infants are both at an increased risk to have type 2 diabetes among schoolchildren in Taiwan. Diabetes Care.

[13] Muktabhant B., Lawrie T.A., Lumbiganon P., Laopaiboon M. (2015). Diet or exercise, or both, for preventing excessive weight gain in pregnancy. Cochrane Database Syst. Rev..

[14] Pauley A.M., Hohman E., Savage J.S., Rivera D.E., Guo P., Leonard K.S., Downs D.S. (2018). Gestational Weight Gain Intervention Impacts Determinants of Healthy Eating and Exercise in Overweight/Obese Pregnant Women. J. Obes..

[15] Downs D.S. (2016). Obesity in Special Populations: Pregnancy. Prim. Care.

[16] Downs D.S., Savage J.S., Rauff E.L. (2014). Falling Short of Guidelines? Nutrition and Weight Gain Knowledge in Pregnancy. J. Women’s Heal. Care.

[17] Dong Y., Rivera D.E., Downs D.S., Savage J.S., Thomas D.M., Collins L.M. Hybrid Model Predictive Control for Optimizing Gestational Weight Gain Behavioral Interventions. Proceedings of the American Control Conference.

[18] Symons Downs D., Savage J.S., Rivera D.E., Smyth J.M., Rolls B.J., Hohman E.E., McNitt K.M., Kunselman A.R., Stetter C., Pauley A.M. (2018). Individually Tailored, Adaptive Intervention to Manage Gestational Weight Gain: Protocol for a Randomized Controlled Trial in Women with Overweight and Obesity. JMIR Res. Protoc..

[19] Collins L.M., Murphy S.A., Strecher V. (2007). The multiphase optimization strategy (MOST) and the sequential multiple assignment randomized trial (SMART): New methods for more potent eHealth interventions. Am. J. Prevent. Med..

[20] Christian P., Mullany L.C., Hurley K.M., Katz J., Black R.E. (2015). Nutrition and maternal, neonatal, and child health. Semin. Perinatol..

[21] Livingstone M.B., Black A.E. (2003). Markers of the validity of reported energy intake. J. Nutr..

[22] Sui Z., Cramp C., Moran L.J., McNaughton S.A., Deussen A.R., Grivell R.M., Dodd J.M. (2018). The characterisation of overweight and obese women who are under reporting energy intake during pregnancy. BMC Pregnancy Childbirth.

[23] Guo P., Rivera D.E., Savage J.S., Hohman E.E., Pauley A.M., Leonard K.S., Symons Downs D. (2018). System identification approaches for energy intake estimation: Enhancing interventions for managing gestational weight gain. IEEE Trans. Control Syst. Technol..

[24] Cappelleri J.C., Bushmakin A.G., Gerber R., Leidy N.K., Sexton C.C., Lowe M.R., Karlsson J. (2009). Psychometric analysis of the Three-Factor Eating Questionnaire-R21: Results from a large diverse sample of obese and non-obese participants. Int. J. Obes..

[25] Stunkard A.J., Messick S. (1985). The three-factor eating questionnaire to measure dietary restraint, disinhibition and hunger. J. Psychosom. Res..

[26] Stunkard A.J., Messick S. (1988). Eating Inventory Manual.

[27] Lowe M.R., Kral T.V. (2006). Stress-induced eating in restrained eaters may not be caused by stress or restraint. Appetite.

[28] Lindroos A.-K., Mathiassen M.E., Bengtsson C., Lindroos A., Lissner L., Karlsson J., Sullivan M., Sjöström L. (1997). Dietary intake in relation to restrained eating, disinhibition, and hunger in obese and nonobese Swedish women. Obes. Res..

[29] Hainer V., Kunešová M., Bellisle F., Parizkova J., Braunerova R., Wagenknecht M., Lajka J., Hill M., Stunkard A. (2006). The eating inventory, body adiposity and prevalence of disease in a quota sample of Czech adults. Int. J. Obesity (Lond.).

[30] Williamson D.A., Lawson O.J., Brooks E.R., Wozniak P.J., Ryan D.H., Bray G.A., Duchmann E.G. (1995). Association of body mass with dietary restraint and disinhibition. Appetite.

[31] Hays N.P., Bathalon G.P., McCrory M.A., Roubenoff R., Lipman R., Roberts S.B. (2002). Eating behavior correlates of adult weight gain and obesity in healthy women aged 55–65 y. Am. J. Clin. Nutr..

[32] Smith C.F., Geiselman P.J., Williamson D.A., Champagne C.M., Bray G.A., Ryan D.H. (1998). Association of dietary restraint and disinhibition with eating behavior, body mass, and hunger. Eat. Weight Disord. Ewd.

[33] Foster G.D., Wadden T.A., Swain R.M., Stunkard A.J., Platte P., Vogt R.A. (1998). The eating inventory in obese women: Clinical correlates and relationship to weight loss. Int. J. Obes..

[34] Lauzon-Guillain B., Basdevant A., Romon M., Karlsson J., Borys J.M., Charles M.A. (2006). Is restraint eating a risk factor for weight gain in a general population?. Am. J. Clin. Nutr..

[35] Lowe M.R., Annunziato R.A., Markowitz J.T., Didie E., Bellace D.L., Riddell L., Maille C., McKinney S., Stice E. (2006). Multiple types of dieting prospectively predict weight gain during the freshman year of college. Appetite.

[36] Olea Lopez A.L., Johnson L. (2016). Associations between Restrained Eating and the Size and Frequency of Overall Intake, Meal, Snack and Drink Occasions in the UK Adult National Diet and Nutrition Survey. PLoS ONE.

[37] Bongers P., Jansen A. (2016). Emotional Eating Is Not What You Think It Is and Emotional Eating Scales Do Not Measure What You Think They Measure. Front. Psychol..

[38] Hill J.O., Wyatt H.R., Reed G.W., Peters J.C. (2003). Obesity and the environment: Where do we go from here?. Science.

[39] Keränen A.-M., Savolainen M.J., Reponen A.H., Kujari M.-L., Lindeman S.M., Bloigu R.S., Laitinen J.H., Teeriniemi A.-M. (2009). The effect of eating behavior on weight loss and maintenance during a lifestyle intervention. Prev. Med..

[40] Mumford S.L., Siega-Riz A.M., Herring A., Evenson K.R. (2008). Dietary restraint and gestational weight gain. J. Am. Diet. Assoc..

[41] Heery E., Wall P.G., Kelleher C.C., McAuliffe F.M. (2016). Effects of dietary restraint and weight gain attitudes on gestational weight gain. Appetite.

[42] Blau L.E., Orloff N.C., Flammer A., Slatch C., Hormes J.M. (2018). Food craving frequency mediates the relationship between emotional eating and excess weight gain in pregnancy. Eat. Behav..

[43] Hutchinson A.D., Charters M., Prichard I., Fletcher C., Wilson C. (2017). Understanding maternal dietary choices during pregnancy: The role of social norms and mindful eating. Appetite.

[44] Lawson O.J., Champagne C.M., Brooks E.R., Howat P.M., Wozniak P.J., Williamson D.A., Delany J.P., Bray G.A., Ryan D.H. (1995). The association of body weight, dietary intake, and energy expenditure with dietary restraint and disinhibition. Obes. Res..

[45] Savage J.S., Hoffman L., Birch L.L. (2009). Dieting, restraint, and disinhibition predict women’s weight change over 6 y. Am. J. Clin. Nutr..

[46] Rivera D.E., Hekler E., Savage J.S., Symons Downs D., Collins L.M. (2018). Intensively adaptive interventions using control systems engineering: Two illustrative examples. Optimization of Behavioral, Biobehavioral, and Biomedical Interventions.

[47] Ajzen I. (1991). The theory of planned behavior. Organ. Behav. Hum. Decis. Process..

[48] Carver C., Scheier M. (1998). On the Self-Regulation of Behavior.

[49] Harris P.A., Taylor R., Thielke R., Payne J., Gonzalez N., Conde J.G. (2009). Research electronic data capture (REDCap)—A metadata-driven methodology and workflow process for providing translational research informatics support. J. Biomed. Inf..

[50] Hadlock F.P., Harrist R.B., Carpenter R.J., Deter R.L., Park S.K. (1984). Sonographic estimation of fetal weight. The value of femur length in addition to head and abdomen measurements. Radiology.

[51] Villar J., Ismail L.C., Victora C.G., Ohuma E., Bertino E., Altman D.G., Lambert A., Papageorghiou A.T., Carvalho M., Jaffer Y. (2014). International standards for newborn weight, length, and head circumference by gestational age and sex: The Newborn Cross-Sectional Study of the INTERGROWTH-21st Project. Lancet.

[52] Black M.H., Sacks D.A., Xiang A.H., Lawrence J.M. (2013). The relative contribution of prepregnancy overweight and obesity, gestational weight gain, and IADPSG-defined gestational diabetes mellitus to fetal overgrowth. Diabetes Care.

[53] Clark M., Ogden J. (1999). The impact of pregnancy on eating behaviour and aspects of weight concern. Int. J. Obes. Relat. Metab. Disord..

[54] Fairburn C.G., Stein A., Jones R. (1992). Eating habits and eating disorders during pregnancy. Psychosom. Med..

[55] Shloim N., Hetherington M.M., Rudolf M., Feltbower R.G. (2015). Relationship between body mass index and women’s body image, self-esteem and eating behaviours in pregnancy: A cross-cultural study. J. Health Psychol..

[56] Slane J.D., Levine M.D. (2015). Association of Restraint and Disinhibition to Gestational Weight Gain among Pregnant Former Smokers. Women’s Health Issues.

[57] Wright C.M., Parkinson K.N., Drewett R.F. (2006). The influence of maternal socioeconomic and emotional factors on infant weight gain and weight faltering (failure to thrive): Data from a prospective birth cohort. Arch. Dis. Child..

[58] Wang M.L., Arroyo J., Druker S., Sankey H.Z., Rosal M.C. (2015). Knowledge, Attitudes and Provider Advice by Pre-Pregnancy Weight Status: A Qualitative Study of Pregnant Latinas with Excessive Gestational Weight Gain. Women Health.

[59] Chang M.W., Nitzke S., Buist D., Cain D., Horning S., Eghtedary K. (2015). I am pregnant and want to do better but I can’t: Focus groups with low-income overweight and obese pregnant women. Matern. Child Health J..

[60] Chang M.W., Nitzke S., Guilford E., Adair C.H., Hazard D.L. (2008). Motivators and barriers to healthful eating and physical activity among low-income overweight and obese mothers. J. Am. Diet Assoc..

[61] Levoy E., Lazaridou A., Brewer J., Fulwiler C. (2017). An exploratory study of Mindfulness Based Stress Reduction for emotional eating. Appetite.

[62] Armitage C.J. (2015). Randomized test of a brief psychological intervention to reduce and prevent emotional eating in a community sample. J. Public Health (Oxf.).

[63] Katterman S.N., Kleinman B.M., Hood M.M., Nackers L.M., Corsica J.A. (2014). Mindfulness meditation as an intervention for binge eating, emotional eating, and weight loss: A systematic review. Eat. Behav..

[64] Conway R., Reddy S., Davies J. (1999). Dietary restraint and weight gain during pregnancy. Eur. J. Clin. Nutr..

[65] Laraia B., Epel E., Siega-Riz A.M. (2013). Food insecurity with past experience of restrained eating is a recipe for increased gestational weight gain. Appetite.

